# S1PR1 suppresses lung adenocarcinoma progression through p-STAT1/miR-30c-5 p/FOXA1 pathway

**DOI:** 10.1186/s13046-024-03230-5

**Published:** 2024-11-18

**Authors:** Yanfei Chai, Hong Xiang, Yuchao Ma, Wei Feng, Zhibin Jiang, Qianjun Zhu, Yingji Chen, Quanjun Liu, Jing Zhang, Jie Ouyang, Peng Gao, Xiao Zhang, Shuhua Chen, Longyu Jin, Hongwei Lu

**Affiliations:** 1 Department of Health Management Center, The Third Xiangya Hospital of Central South University, Changsha, China; 2 Department of Cardiothoracic Surgery, The Third Xiangya Hospital of Central South University, Changsha, China; 3 Department of Thoracic Surgery, The Tenth Affiliated Hospital of Southern Medical University (Dongguan People’s Hospital), Dongguan, Guangdong China; 4 Center for Experimental Medicine, The Third Xiangya Hospital of Central South University, Changsha, China; 5 Department of Biochemistry, School of Life Sciences of Central South University, Changsha, China

**Keywords:** S1PR1, Lung Adenocarcinoma, FOXA1, miR-30c-5p

## Abstract

**Background:**

Sphingosine-1-phosphate receptor 1 (S1PR1) is considered to be closely related to a variety of malignant tumors, but the role and mechanism of S1PR1 in lung adenocarcinoma are not fully understood. In this study, we aim to explore the role and downstream signaling pathways of S1PR1 in the malignant biological functions of lung adenocarcinoma (LUAD).

**Methods:**

Bioinformatics analysis, RT-qPCR, western blot and immunohistochemistry (IHC) were was used to investigate the expression of S1PR1 in LUAD. The prognosis of S1PR1 was also analyzed. CCK-8 assay, colony formation assay, scratch assay, transwell migration and invasion assay, cell adhesion assay were performed to examine the effect of S1PR1 on LUAD. RNA sequencing was employed to analyze the DEGs in LUAD cells overexpressing S1PR1. Enrichment pathway analysis using KEGG, GO, and GSEA was conducted to predict potential signaling pathways and downstream targets. chromatin immunoprecipitation (ChIP) and dual luciferase reporter assay were performed to verify the direct regulation between FOXA1 and the target genes. Then FOXA1 overexpression were performed to functional rescue experiments. miRNA-30c-5p was identified as a microRNA regulating FOXA1 by dual luciferase reporter assay. The downstream signaling pathways of S1PR1 was detected to clarify the specific pathways to regulates miR-30c-5p.

**Results:**

S1PR1 is significantly decreased in LUAD and is positively correlated with the prognosis. Overexpression of S1PR1 inhibits the proliferation, migration, invasion and adhesion function of LUAD cells by suppressing the expression of COL5A1, MMP1, and SERPINE1. FOXA1 is a key transcription factor regulating the expression of MMP1, COL5A1 and SERPINE1. S1PR1 inhibits the expression of FOXA1 through p-STAT1/miR-30c-5p, thereby suppressing the malignant function of LUAD cells.

**Conclusions:**

The expression of S1PR1 is downregulated in LUAD, which is positively correlated with prognosis. S1PR1 regulates the malignant function of LUAD cells by inhibiting the expression of COL5A1, MMP1 and SERPINE1 through the p-STAT1/miR-30c-5p/FOXA1 signaling pathway.

**Supplementary Information:**

The online version contains supplementary material available at 10.1186/s13046-024-03230-5.

## Background

Lung cancer, as the most prevalent malignancy globally, maintains consistently elevated incidence and mortality rates. According to recent statistical data, lung cancer has emerged as the most frequently diagnosed cancer in 2022, accounting for an estimated 2.5 million new cases, which constitutes 12.4% of the total global cancer burden. Moreover, lung cancer remains the leading cause of cancer-related deaths, with an estimated 1.8 million fatalities, representing 18.7% of all cancer deaths. It’s noteworthy that among males, lung cancer dominates both in incidence and mortality, whereas it holds the second position among females, trailing only breast cancer [1].

Lung cancer can be classified into two primary subcategories: non-small cell lung cancer (NSCLC) and small cell lung cancer. Among these, NSCLC comprises the overwhelming majority, accounting for approximately 85% of all cases. Within the NSCLC category, lung adenocarcinoma (LUAD) stands as a significant branch, representing over 60% of NSCLC cases. Alarmingly, the incidence of LUAD has been increasing annually, highlighting the need for further research and heightened awareness regarding this specific subtype of lung cancer [2, 3]. Despite advancements in multimodalities for lung cancer, including minimally invasive surgical techniques, stereotactic radiotherapy, molecular targeted therapy, and immunotherapy, the five-year survival rate for lung cancer remains comparatively low, hovering at approximately 20% [4]. Hence, a profound exploration of the underlying malignant processes in lung cancer, particularly those pertaining to its crucial functions like proliferation, apoptosis, migration, invasion, and adhesion, holds immense significance in elucidating the biological nuances of this disease and devising targeted therapeutic strategies.

Sphingosine-1-phosphate (S1P), a bioactive lipid mediator, engages with S1PR1 to activate the Gi protein, subsequently triggering pivotal signaling cascades encompassing the Ras/ERK, PI3K/AKT, and STAT3 pathways [5]. S1PR1 has been implicated in fostering migration, invasion, proliferation, and angiogenesis in an array of malignancies, including bladder cancer [6], esophageal cancer [7], ovarian cancer [8], renal cancer [9], colorectal cancer [10], breast cancer [11], and lung cancer [11]. However, contrasting findings indicate that S1PR1 may also modulate tumor vascular normalization and maintain vascular permeability, thereby exerting inhibitory effects on tumor growth and metastasis [12, 13].

Studies have revealed that S1PR1 in lung cancer tissues exhibits a pronounced downward trajectory compared to normal lung tissues, and this reduced expression is intimately associated with an unfavorable prognosis for lung cancer patients [14, 15]. This finding implies that S1PR1 may contribute to changes in lung cancer cell functionality, thereby influencing the initiation and progression of lung cancer. However, the current understanding of the precise role and underlying mechanisms of S1PR1 in lung cancer remains incomplete.

Therefore, in this study, we analyzed the differential expression of S1PR1 in lung cancer tissues compared to normal lung tissues using database analysis followed by experimental validation. We also investigated the functional role of S1PR1 by modulating its expression in LUAD cells, aiming to further elucidate the specific regulatory mechanisms involved.

## Methods

### TCGA and GEO data analysis

RNA sequencing (RNA-Seq) data in LUAD was thoroughly investigated using datasets from The Cancer Genome Atlas (TCGA) and the Gene Expression Omnibus (GEO).

Specifically, RNA-Seq data (counts format) with corresponding clinical data of LUAD patients were retrieved from the Genomic Data Commons Data Portal (https://portal.gdc.cancer.gov/). Furthermore, expression data for GEO series including GSE116959, GSE19188, GSE19804, GSE30219, GSE32863, GSE40791, GSE43458, GSE75037, and GSE72094 were downloaded from the GEO database (https://www.ncbi.nlm.nih.gov/geo/). Information of the datasets are present in Table S1.

The “limma” package was employed to identify differentially expressed genes (DEGs) between tumor and normal tissues. Genes with |Log2 fold change (FC)|> 1.5 and a Benjamini–Hochberg adjusted *p*-value < 0.01 were defined as DEGs.

### Gene Ontology (GO) functional and Kyoto Encyclopedia of Genes and Genomes (KEGG) pathway enrichment analyses

To determine the biological annotations of robust DEGs, GO functional and KEGG pathway enrichment analyses were conducted and visualized through the “clusterProfiler” package [16]. An adjusted p-value < 0.05 was considered statistically significant difference.

### Patients and clinical samples

A total of twelve primary tumor tissues and their corresponding adjacent normal tissues were procured from patients diagnosed with LUAD at the Third Xiangya Hospital of Central South University. The conduct of this study was authorized and approved by the Human Research Ethics Committee of the Third Xiangya Hospital of Central South University. The clinical and pathological information of the patients were shown in Table S2.

### Cell lines and cell culture

The human LUAD cell lines A549, H1975, and H838, the normal epithelial cell lines BEAS-2B and human embryonic kidney cell 293T, were purchased from Procell (Wuhan, China). A549, H1975, and H838 were cultured in RPMI-1640 medium (Procell, China), while the BEAS-2B and 293T cell lines were maintained in DMEM medium (Procell, China). All cell lines were supplemented with 10% fetal bovine serum (FBS; Procell, China) and 1% penicillin/streptomycin (Sciencell, USA), incubated at 37 °C in 5% CO2.

### Vector construction and cell transfection

The human full-length S1PR1 cDNA was cloned into the Ubi-MCS-3FLAG-CBh-gcGFP-IRES-puromycin lentiviral vector (GV492, Genechem, China). Additionally, the short hairpin RNA (shRNA) targeting human S1PR1 was inserted into the hU6-MCS-Ubiquitin-EGFP-IRES-puromycin lentiviral vector (GV248, Genechem, China). shRNA target sequences for S1PR1 were sh-S1PR1#1: GACAAGGAGAACAGCATTAAA; sh-S1PR1#2: CGCTGCTCAAGACCGTAATTA.

The human full-length FOXA1 cDNA was cloned into the CMV-enhancer-MCS-SV40-puromycin vector (GV712, Genechem, China). Furthermore, the binding site activity of FOXA1 and the 3’UTR regions of human COL5A1, MMP1, and SERPINE1 were cloned into the MCS-firefly_Luciferase vector (Genechem, China).

The miR-30c-5p mimic, miR-30c-5p inhibitor, and their respective negative controls were synthesized and purified by RiboBio (Guangzhou, China). For plasmid transfection, Lipofectamine 3000 reagent (Invitrogen, USA) was utilized according to the manufacturer’s protocol. Stable cell lines were selected using 2 μg/mL puromycin for 48 h (Sigma-Aldrich, USA). Subsequently, the puromycin concentration was reduced to a maintenance level (1/4 to 1/2 of the screening concentration) and continued selecting and expanding the stable cell lines. RNA extraction and Real‑time quantitative PCR (RT-qPCR) assays.

Total RNA was isolated from the cells employing the TRIzol Reagent (Invitrogen, USA). For mRNA analysis, 1 μg of RNA was reverse transcribed into cDNA using the Evo M-MLV RT Mix Kit (Accurate Biology, China), following the manufacturer’s recommended protocol. For the analysis of microRNAs, RNA was reverse transcribed into cDNA utilizing the miRNA 1st Strand cDNA Synthesis Kit (Accurate Biology, China), strictly adhering to the manufacturer’s instructions.

RT-qPCR was conducted using the SYBR Green Premix qPCR Kit (Accurate Biology, China) on the Roche LightCycler 480 II instrument (Roche, Basel, Switzerland). The primer sequences utilized in this study are listed in Table S3.

### Western blot

The tissues and cells were lysed using RIPA buffer (Beyotime, China), and the protein concentration in the supernatant was determined using the bicinchoninic acid (BCA) assay (Beyotime, China). Total protein was then separated via sodium dodecyl sulfate–polyacrylamide gel electrophoresis (SDS-PAGE) and transferred onto a polyvinylidene fluoride (PVDF) membrane (Millipore, USA).

The membrane was blocked with 5% non-fat milk in Tris-buffered saline with Tween 20 (TBST) for 1.5 h at room temperature. Subsequently, the membrane was incubated with primary antibodies overnight at 4 °C. Following this, the membrane was incubated with secondary antibodies for 2 h at room temperature. Finally, the proteins were visualized using a chemiluminescence kit (Thermo Scientific, USA). Antibodies used in this procedure were shown in Table S4.

### Immunohistochemistry

Four-micrometer-thick tissue sections underwent a deparaffinization and rehydration procedure, following which antigen retrieval was conducted. Subsequently, endogenous peroxidase activity was blocked to prevent non-specific staining. The slides were then incubated with primary antibodies specific to the target protein, followed by incubation with appropriate secondary antibodies. After this, the slides were incubated with diaminobenzidine. Finally, the slides were counterstained with hematoxylin to provide contrast and enhance visualization. Antibodies used in this procedure were shown in Table S5.

The intensity of the immunostaining was qualitatively assessed and categorized into four distinct groups: negative (score = 0), weak (score = 1), moderate (score = 2), and strong (score = 3). Additionally, the percentage of positively stained cells was also quantified and classified into four categories: 0–25% (score = 1), 26–50% (score = 2), 51–75% (score = 3), and 76–100% (score = 4). The final IHC score was determined by the multiplying the two scores.

### Cell counting kit-8 assay

In the experimental protocol, 2000 cells were seeded onto 96-well plates and allowed to adhere overnight. Subsequently, CCK-8 reagent (Biosharp, China) was added into each well at a 1:10 dilution. The cells were then incubated for an hour, enabling the CCK-8 reagent to interact with the cells. Following this incubation period, the 450 nm absorbance was measured using a microplate reader (Bio-Rad, Model 680, USA), which provided a quantitative assessment of cell viability. The entire experimental procedure was repeated at least three times.

### Colony formation assay

Single-cell suspensions were seeded into 6-well plates, with approximately 500 cells per well. Following a culture period of 10 to 14 days, ocular cell clusters had formed. The cells were fixed using 4% formaldehyde and subsequently stained with 0.1% crystal violet. A colony was defined as nonoverlapping group of at least 50 cells. Colonies were counted manually by light microscopy. Each experiment was conducted in triplicate to ensure reproducibility and statistical reliability.

### Wound healing assay

5 × 10^5^ LUAD cells were plated into 6-well plates and incubated until a 100% growth. Then, a 200µL pipette tip were used to generate linear scratches. Next, cells were washed twice with PBS. Then, serum-free medium was added to the well [17]. Images were captured at 0 h and 24 h after wounding. Images were analyzed using ImageJ software.

### Transwell migration and invasion assays

For the migration assay, chambers were present in a 24-well culture table with 600 μL RPMI-1640 containing 10% FBS prepared at the bottom. Then, 5 × 10^4^ cells in 100 μL serum-free medium were added into the upper layer [17, 18]. After 24 h, migrated cells in the lower layer were fixed in 4% formaldehyde and stained with 0.1% crystal violet. For the invasion assay pre-colded matrigel (Corning, USA) were added into the upper layer. Then 5 × 10^4^ cells were added to the chamber after coating.

### Cell adhesion assay

5 × 10^4^ Cells in 100 μL serum-free medium were added into 96-well plates which were precoated with fibronectin (Sigma). Plates were incubated for 1 h at 37 °C, then nonadherent cells were removed by a gentle washing three times with PBS, and the remaining cells were fixed in 4% formaldehyde and stained with 0.1% crystal violet. Photos were captured after drying.

### Animal experiments

The research protocol was approved and the mice were maintained according to the Institutional Guidelines of the Animal Ethics Committee of Central South University. Female BALB/c nude mice were purchased from Hunan SLAC Experimental Animal Company. For subcutaneous tumor xenograft mouse model, approximately 1 × 10^7^ A549 cells in 100μL medium were implanted into the posterior flank of nude mice (*n* = 5 per group). The tumor size was measured every 3 days using calipers and tumor volumes were calculated according to the standard formula. V (mm^3^) = (length × width^2^)/2. All mice were sacrificed after 21 days, and the tumor tissues were separated, weighed, and photographed, followed by H&E, Masson’s and IHC staining.

For lung metastasis experiments, cells were suspended in PBS, and mixed with Matrigel (Corning, USA) at a 1:1 ratio. 2 × 10^6^ cells in 100 μL mixture were injected into the tail vein (*n* = 5 per group). Eight weeks after injection, the mice were euthanized, and the lungs were excised for imaging. Subsequent analyses included H&E staining, as well as Masson’s trichrome staining.

### H&E and Masson’s trichrome staining

Fresh tissues were fixed in 4% paraformaldehyde for 24 h. Following fixation, the tissues were dehydrated, embedded in paraffin, and sectioned into 4-μm slices. The paraffin sections underwent the following immersion procedures: xylene for 10 min (repeated three times), 100% ethanol for 5 min, 95% ethanol for 5 min, 85% ethanol for 5 min, 75% ethanol for 5 min, and then rinsed with tap water. The sections were subsequently placed in hematoxylin solution for 3 min, rinsed with tap water for 3 min, dipped twice in 1% acid alcohol, and rinsed with cold running tap water until the tissue sections turned blue. Next, the sections were placed in 5% ethanol for 5 min and 95% ethanol for 5 min, stained with eosin dye for 5 min, and then dehydrated and mounted with neutral gum. Masson’s trichrome staining was performed according to the manufacturer’s instructions (Solarbio, China).

### Chromatin immunoprecipitation (ChIP)

Cells were cross-linked with 1% formaldehyde solution, neutralized by 1.25 M glycine, then lysed and sonicated. Then the lysates were incubated at 4 °C overnight with the target-specific antibody FOXA1 (Cell Signaling Technology, USA) or STAT1 (Cell Signaling Technology, USA), or negative control of normal Rabbit IgG. The immunoprecipitated complexes was precleared with ChIP Grade protein A/G Magnetic beads for 2 h. Then DNA was eluted from immunoprecipitated complexes, reverse cross-linked, and purified. High-quality ChIP DNA were used for RT-qPCR as former procedure. The antibodies were listed in Table S6, and primer sequences in this procedure were listed in Table S7.

### Dual - luciferase reporter assay

The binding sites of miRNAs were predicted by TargetScan (http://www.targetscan.org/). The different 3’‐UTR sequences were synthesized by Genechem and added into the vector to generate reporter plasmids. 293T cells were seeded into 96‐well plates and transfected with the indicated reporter plasmid or miR‐30c‐5p mimics using lipofectamine 3000 reagent. Then, luciferase activity was assessed after post-transfection for 48 h using the Dual‐Luciferase Assay System (Vazyme, China) according to the manufacturer’s instructions.

### RNA-seq

Total RNA was isolated from the A549 cells using Trizol following the manufacturer’s protocol (Invitrogen). The sample quality control, library construction and RNA sequencing were performed and analyzed by BGI tech (Shenzhen, China). “DESeq2” package was used to analyze DEGs between A549 cells with S1PR1 overexpressing and negative controls. t-SNE analysis was conducted to investigate the distribution of cells using the “Rtsne” package. GO and KEGG enrichment analyses were performed as previously described.

### Protein–protein interaction (PPI) network

The “STRINGdb” package was used to predict the PPI of DEGs in the STRING database (http://string-db.org/) [19] with a confidence cutoff of 0.4. The PPI network was visualized with the Cytoscape software (Version 3.90, http://www.cytoscape.org/). The CytoHubba plugin was used to calculate the scores of each genes and ranked by “RobustRankAggreg” package [20]. DEGs with adjusted *p*-values < 0.05 were identified as hub genes.

### Gene set enrichment analysis (GSEA)

To explore the KEGG pathways associated with changes in A549 cells following S1PR1 overexpression, GSEA was conducted using the “gsKEGG” function in the “clusterProfiler” package. All genes were ranked based on their log2FC, and gene sets with a false discovery rate (q-value) of less than 0.05 were considered statistically significant.

### Statistical analysis

The results are represented as the means ± SD of at least three independent experiments of biological replicates. Student’s t-test or two-way ANOVA was used to compare the differences between groups, as appropriate. Log-rank test and Kaplan–Meier analysis was used to assess the survival difference. *P* < 0.05 was considered statistically significant. Statistical analysis was performed with GraphPad Prism 9.0. and R software (version 4.2.2).

## Results

### S1PR1 was downregulated in LUAD and Low expression of S1PR1 was related with poorer prognosis

**Fig. 1:**
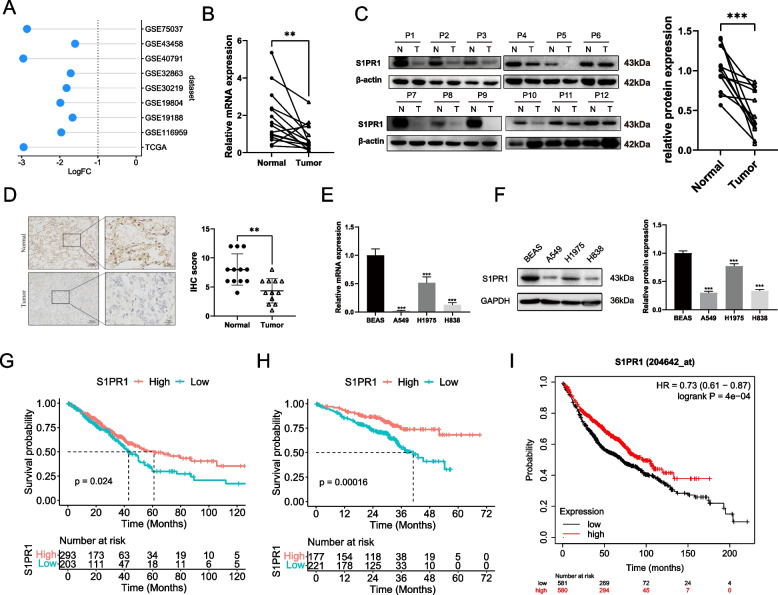
S1PR1 was downregulated in LUAD and Low expression of S1PR1 was related with poorer prognosis. **A** Differential expression of S1PR1 between normal and tumor tissues of LUAD patients in nine datasets. **B** mRNA expression levels of S1PR1 in tumor and normal tissues of LUAD patients. **C** Protein expression levels of S1PR1 in tumor and normal tissues of LUAD patients. **D** IHC score levels of S1PR1 in tumor and normal tissues of LUAD patients. **E** mRNA expression levels of S1PR1 in normal lung cell BEAS-2B and LUAD cells. **F** Protein expression levels of S1PR1 in BEAS-2B and LUAD cells. **G-I**, Kaplan–Meier curves for OS in LUAD patients with high and low expression of S1PR1 in the TCGA cohort(**G**), GSE72094(**H**) and Kaplan–Meier plotter(**I**). **P < 0.01, ***P < 0.001

To elucidate the disparate expression of S1PR1 between tumor and normal tissues in LUAD patients and its association with clinical outcomes, we conducted a comprehensive analysis of the RNA expression matrix. Using the TCGA and GEO databases, the “limma” package was employed to screen DEGs between tumor and normal tissues of LUAD patients in TCGA as well as eight GEO datasets. The findings revealed a significant downregulation of S1PR1 in LUAD tissues compared to normal lung tissues across all nine datasets(Fig. 1A, Figure S1).

To validate the bioinformatics analysis findings, we further examined surgical specimens from LUAD patients. The results confirmed a significant reduction in S1PR1 expression in LUAD tissues compared to normal lung tissues, both at the mRNA and protein levels (Fig. 1B-C). Furthermore, IHC detection corroborated this finding, demonstrating a significantly lower expression of S1PR1 in LUAD tissues compared to normal tissues (Fig. 1D). These concordant findings strengthen the conclusion that S1PR1 expression is diminished in LUAD. We also observed decreased expression of S1PR1 at both the mRNA (Fig. 1E) and protein (Fig. 1F) levels in LUAD cell lines A549, H1975 and H838, compared to normal lung epithelial cells BEAS-2B. These results indicate that S1PR1 is downregulated in LUAD and may play an important role in LUAD.

Then, we investigate association between S1PR1 and the prognosis of LUAD patients in the TCGA and GSE72094 datasets. Survival analysis revealed that, within the TCGA database, patients with low S1PR1 expression displayed significantly poorer prognosis compared to those with high S1PR1 expression (Fig. 1G). This finding was further validated using the GSE72094 (Fig. 1H). Additionally, the results using the Kaplan–Meier plotter concurred with the results obtained from the aforementioned two databases (Fig. 1I). These consistent results suggest a positive correlation between S1PR1 expression and the prognosis of LUAD, implying a potential role of S1PR1 in inhibiting the progression of LUAD.

Based on the prognostic analysis, we stratified LUAD patients in the TCGA database into two groups: high S1PR1 expression and low S1PR1 expression. A total of 1882 DEGs had been identified between these two groups (Figure S2). Further, GO functional enrichment revealed that these DEGs were primarily associated with extracellular matrix (ECM)-related functions, encompassing collagen-containing ECM, focal adhesion, extracellular matrix organization, and extracellular matrix structural constituent. Additionally, KEGG pathway enrichment indicated that the DEGs were enriched in cell adhesion molecules, focal adhesion, ECM-receptor interaction, PI3K-AKT signaling pathway, chemokine signaling pathway, and other relevant pathways (Figure S3). These results suggest a potential intimate relationship between S1PR1 and functional alterations in the ECM.

### S1PR1 inhibits the malignant biological function of LUAD

**Fig. 2:**
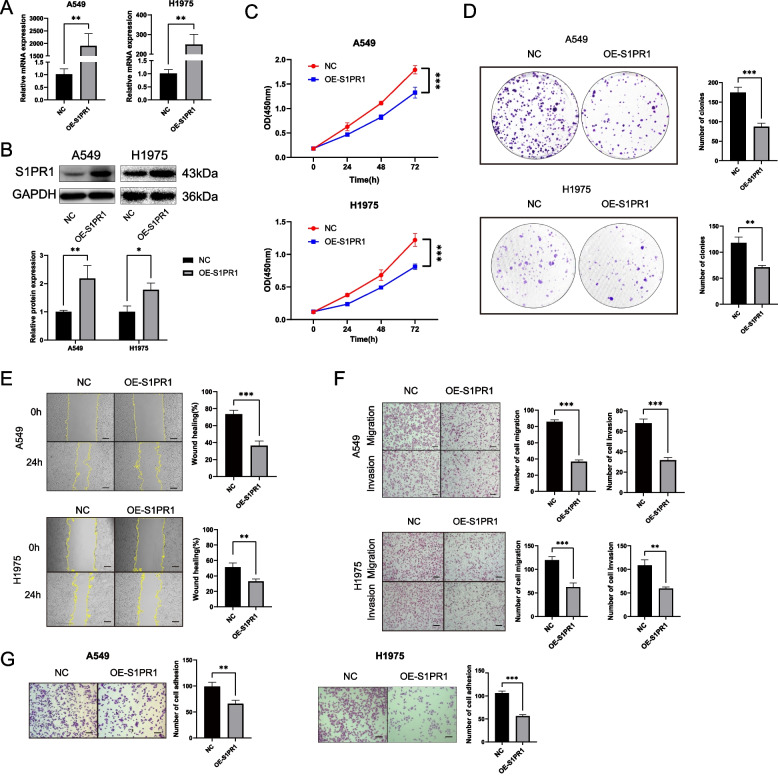
S1PR1 inhibits the proliferation, migration, invasion and adhesion of LUAD cells. **A** mRNA expression levels and **B** protein levels of S1PR1 in A549 and H1975 cells with stable overexpression of S1PR1. **C** Effect of S1PR1 overexpression on the proliferation ability of A549 and H1975 cells by CCK-8 assay. **D** Effect of S1PR1 overexpression on the colonies formation of A549 and H1975 cells by colony formation assay. **E** Wound healing assay showed S1PR1 overexpression inhibited migration ability of A549 and H1975 cells. Scale bar = 300 μm. **F** Effect of S1PR1 overexpression on the migration and invasive ability of A549 and H1975 cells by transwell migration and invasive assay. Scale bar = 150 μm. **G** Effect of S1PR1 overexpression on the adhesion ability of A549 and H1975 cells by adhesion assay. Scale bar = 150 μm. *P < 0.05, **P < 0.01 and ***P < 0.001

To elucidate the impact of S1PR1 on the functional phenotype of LUAD cell lines, we modulated S1PR1 expression using lentiviral vectors encoding either S1PR1-overexpressing or shRNA. According to our previous findings, we transduced A549 and H1975 cells with S1PR1-overexpressing lentivirus (Fig. 2 A-B). Additionally, to achieve knockdown of S1PR1, we transduced shRNA lentiviral vectors (sh-S1PR1#1, sh-S1PR1#2) into H1975 cells, which exhibited the highest endogenous S1PR1 expression (Figure S4 A-B).

We investigate the role of S1PR1 on the malignant function of LUAD cells, such as proliferation, migration, invasion and adhesion. CCK-8 assay revealed a marked reduction in the proliferation rate of A549 and H1975 cells overexpressing S1PR1 (Fig. 2C). Consistently, the colony formation assay demonstrated a significant decrease in the number of single-cell clones formed after S1PR1-overexpressing (Fig. 2D). Conversely, knockdown of S1PR1 in H1975 cells led to a significant enhancement in proliferative and colony-forming capacity (Figure S4 C-D). Would healing assay and transwell migration assay demonstrated that overexpression of S1PR1 significantly suppressed the migration of A549 and H1975 cells (Fig. 2E-F), while knockdown of S1PR1 in H1975 cells conspicuously augmented their migration potential (Figure S4E-F). Furthermore, S1PR1 significantly reduced the number of A549 and H1975 cells that traversed the matrigel-coated membrane (Fig. 2F). Conversely, knockdown of S1PR1 significantly increased the invasion capacity (Figure S4F). Cell adhesion assay revealed a substantial reduction in the number of A549 and H1975 cells adhering to fibronectin following S1PR1 overexpression (Fig. 2G), while knockdown of S1PR1 promoted the adhesion ability in H1975 cells (Figure S4G). Collectively, these findings indicate that S1PR1 suppress the malignant phenotypes of LUAD cells in vitro.

### S1PR1 affects LUAD function by regulating extracellular matrix through COL5A1、MMP1、SERPINE1

**Fig. 3:**
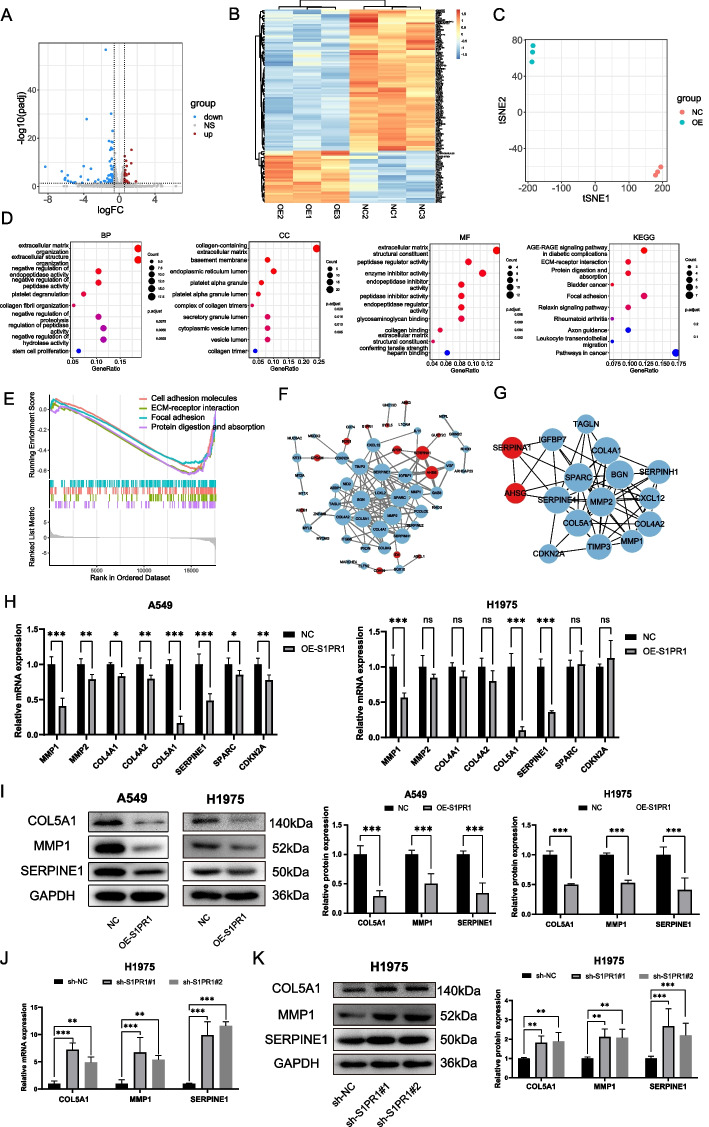
S1PR1 inhibits the malignant function of LUAD by regulating extracellular matrix through COL5A1, MMP1, SERPINE1. **A** Volcano plot and **B** heatmap displayed the DEGs between A549 cells with NC or OE-S1PR1. **C** t-SNE analysis of the DEGs between A549 cells with NC or OE-S1PR1. **D** GO function and KEGG pathway enrichment analysis of the DEGs. **E** GSEA enrichment analysis of KEGG pathway between A549 cells with NC or OE-S1PR1. **F** PPI network construction of the DEGs between A549 cells with NC or OE-S1PR1. **H** Hub genes screened using cytoHubba plugin in cytoscape. **H** mRNA expression levels of selected hub genes detected by RT-qPCR in A549 and H1975 cells with S1PR1 overexpression. **I** protein levels of COL5A1, MMP1, and SERPINE1 detected by western blot in A549 and H1975 cells with S1PR1 overexpression. **J** mRNA expression levels of COL5A1, MMP1, and SERPINE1 detected by RT-qPCR in H1975 cells with S1PR1 knockdown. (K) Protein levels of COL5A1, MMP1, and SERPINE1 detected by western blot in H1975 cells with S1PR1 knockdown. ns, not significant, *P < 0.05, **P < 0.01 and ***P < 0.001

To elucidate the precise mechanism underlying S1PR1’s regulation of LUAD function, RNA sequencing was performed to identify DEGs between A549 cells overexpressing S1PR1 and negative control cells. The Volcano plot (Fig. 3A) and heatmap (Fig. 3B) revealed 77 downregulated genes and 26 upregulated genes after S1PR1 overexpression. t-SNE analysis demonstrated that these DEGs could effectively discriminate A549 cells overexpressing S1PR1 from control cells (Fig. 3C). GO and KEGG analysis found these genes were primarily enriched in functions and pathways related to ECM. As revealed by GO analysis, DEGs enriched in collagen-containing ECM, focal adhesion, ECM organization, and ECM structural constituent. KEGG pathway analysis revealed that the DEGs were enriched in ECM-receptor interaction, protein absorption and digestion, and focal adhesion pathways (Fig. 3D, Table S8).GSEA analysis also demonstrated that, in terms of KEGG pathways, cell adhesion molecules, ECM-receptor interaction, protein absorption and digestion, and focal adhesion, were significantly enriched and exhibited a downward trend (Fig. 3E). Thus, we hypothesized that S1PR1 may participates in the regulation of ECM, thereby modulating the functional properties of LUAD cells.

We further elaborated a PPI network using DEGs, which contain 57 nodes and 137 edges (Fig. 3F). Leveraging the “cytoHubba” plugin in Cytoscape, along with the RRA algorithm, we identified 16 hub genes with adj.*p* < 0.01 (Fig. 3G). These genes exhibit a profound association with ECM and encompass proteins such as AHSG, BGN, MMP1, MMP2, COL4A1, COL4A2, COL5A1, SERPINE1, SERPINA1, SERPINH1, SPARC, TIMP3, and CDKN2A. These proteins play critical roles in ECM degradation, remodeling, and cross-linking, and have been implicated in facilitating tumor progression across various types of cancer. Our analysis revealed that S1PR1 could negatively regulate these genes, suggesting that S1PR1 fulfills a tumor-suppressive role by inhibiting the ECM.

RT-qPCR revealed a marked downregulation of MMP1, MMP2, COL4A1, COL4A2, COL5A1, SPARC, SERPINE1, and CDKN2A in A549 cells overexpressing S1PR1, which concurred with the transcriptome sequencing results. Similarly, in H1975 cells, S1PR1 overexpression led to a significant reduction in COL5A1, MMP1, and SERPINE1. Meanwhile, MMP2, COL4A1, and COL4A2 also showed a decreasing trend but this difference did not reach statistical significance, and SPARC and CDKN2A remained unchanged (Fig. 3H).

In alignment with RT-qPCR, western blot showed that COL5A1, MMP1, and SERPINE1 was significantly reduced in both A549 and H1975 cells upon S1PR1 overexpression (Fig. 3I). Furthermore, in H1975 cells, knockdown of S1PR1 led to a significant upregulation in mRNA and protein expressions of COL5A1, MMP1, and SERPINE1 (Fig. 3J-K). These findings suggest that S1PR1 inhibits the malignant biological processes of LUAD by downregulating the expression of COL5A1, MMP1, and SERPINE1.

### S1PR1 regulates COL5A1, MMP1 and SERPINE1 through the transcription factor FOXA1

**Fig. 4:**
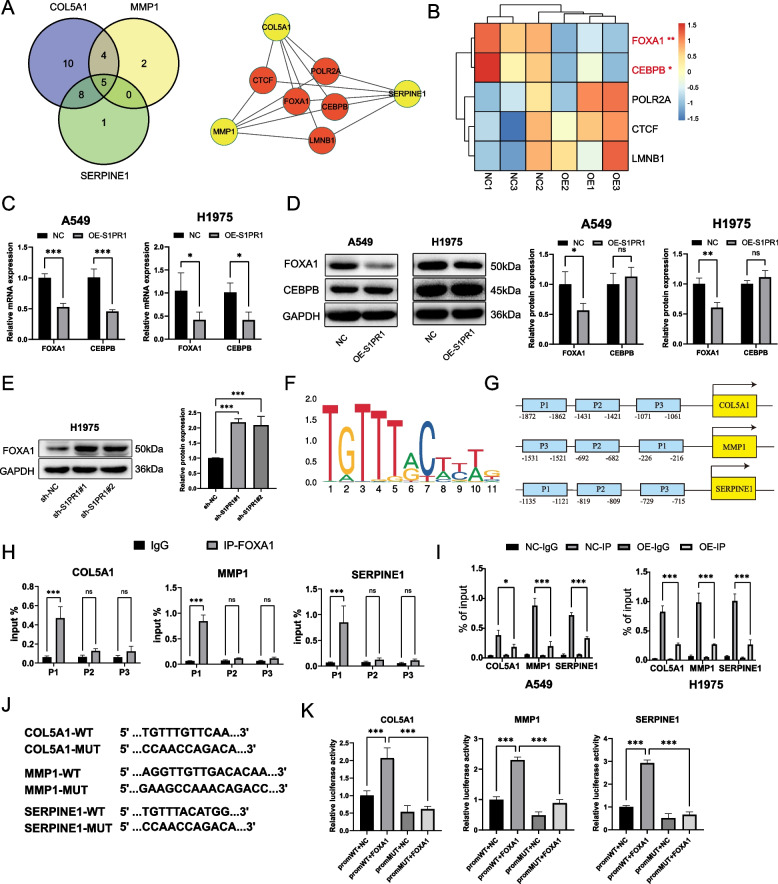
S1PR1 regulates COL5A1, MMP1 and SERPINE1 through the transcription factor FOXA1. **A** Potential transcription factors that regulates the expression of COL5A1, MMP1, and SERPINE1 in the hTFtarget database. **B** Heatmap of the transcription factors in RNA sequencing data. **C** mRNA levels of FOXA1 and CEBPB detected by RT-qPCR in A549 and H1975 cells with S1PR1 overexpression. **D** Protein levels of FOXA1 and CEBPB detected by western blot in A549 and H1975 cells with S1PR1 overexpression. **E** Protein levels of FOXA1 detected by western blot in H1975 cells with S1PR1knockdown. **F** The motif of FOXA1 predicted by JASPAR database. **G** The potential binding sites of FOXA1 in promoters of COL5A1, MMP1 and SERPINE1 were predicted by JASPAR database. **H** ChIP-qPCR of FOXA1 binding to promoters of COL5A1, MMP1 and SERPINE1 in A549 cells. **I** ChIP-qPCR of FOXA1 binding to promoters(P1) of COL5A1, MMP1 and SERPINE1 in A549 and H1975 cells with S1PR1 overexpression. **J** Predicted binding sites of FOXA1 in promoters of COL5A1, MMP1 and SERPINE1 and matched mutant sequences. **H** Luciferase reporter assay showed the luciferase activity of FOXA1 on the wildtype and mutant promoter of COL5A1, MMP1 and SERPINE1. ns, not significant, *P < 0.05, **P < 0.01 and ***P < 0.001

To elucidate the complex mechanisms through which S1PR1 regulates the expression of COL5A1, MMP1, and SERPINE1, we performed a comprehensive search for transcription factors involved in their regulation using the hTFtarget database. Our findings revealed that in lung cancer tissues or cells, five transcription factors—FOXA1, CEBPB, CTCF, LMNB1, and POLR2A—concurrently regulate the transcription of COL5A1, MMP1, and SERPINE1 (Fig. 4A). RNA sequencing demonstrated a notable downregulation in FOXA1 and CEBPB following overexpression of S1PR1, while CTCF, LMNB1, and POLR2A remained unchanged (Fig. 4B). RT-qPCR showed a significant downregulation in mRNA expression of FOXA1 and CEBPB in both A549 and H1975 cells following S1PR1 overexpression (Fig. 4C). Western blot revealed that S1PR1 overexpression inhibited the expression of FOXA1 in both A549 and H1975 cells, while CEBPB did not change (Fig. 4D). Intriguingly, knockdown of S1PR1 in H1975 cells led to a significant upregulation in FOXA1 (Fig. 4E). These findings indicated that S1PR1 inhibit the expression of FOXA1, potentially influencing the expression of COL5A1, MMP1, and SERPINE1.

Transcription factors regulate gene expression by binding to specific sites within the promoters of target genes. Utilizing the JARSPAR database, we predicted the binding sites of FOXA1 on the promoter of COL5A1, MMP1, and SERPINE1 (Table S9). Figure 4F illustrates the motif of FOXA1 on the promoters of target genes. Based on these predictions, we subsequently constructed primers of the binding sites at the promoters of COL5A1, MMP1, and SERPINE1(Fig. 4G). ChIP assay revealed that FOXA1 can directly bind to the specific site P1 in the promoter of COL5A1, MMP1, and SERPINE1. In contrast, the binding capacity to other sites (P2 and P3) did not significantly differ from IgG control (Fig. 4H). Moreover, S1PR1 overexpression decreased the FOXA1 levels at the promoter region of COL5A1, MMP1, and SERPINE1 (Fig. 4I), suggesting that S1PR1 inhibits the expression of COL5A1, MMP1, and SERPINE1 through downregulation of FOXA1. Subsequently, we constructed wild-type and mutant plasmids in the promoter regions of COL5A1, MMP1, and SERPINE1 (Fig. 4J). Dual luciferase reporter assay showed that FOXA1 bound to the promoters of COL5A1, MMP1, and SERPINE1, leading to a significant increasing of luciferase activity, and the luciferase activity was notably reduced when the binding sites were mutated (Fig. 4K). These findings further corroborate that FOXA1 promotes the transcription of COL5A1, MMP1, and SERPINE1 by binding to their promoter regions.

Therefore, we identified that FOXA1 is a key transcription factor in regulating the expression of COL5A1, MMP1, and SERPINE1, and S1PR1 may suppress the malignant biological processes of LUAD cells by inhibiting FOXA1.

### FOXA1 reversed the inhibitory effect of S1PR1 on LUAD cells in vitro and in vivo

**Fig. 5:**
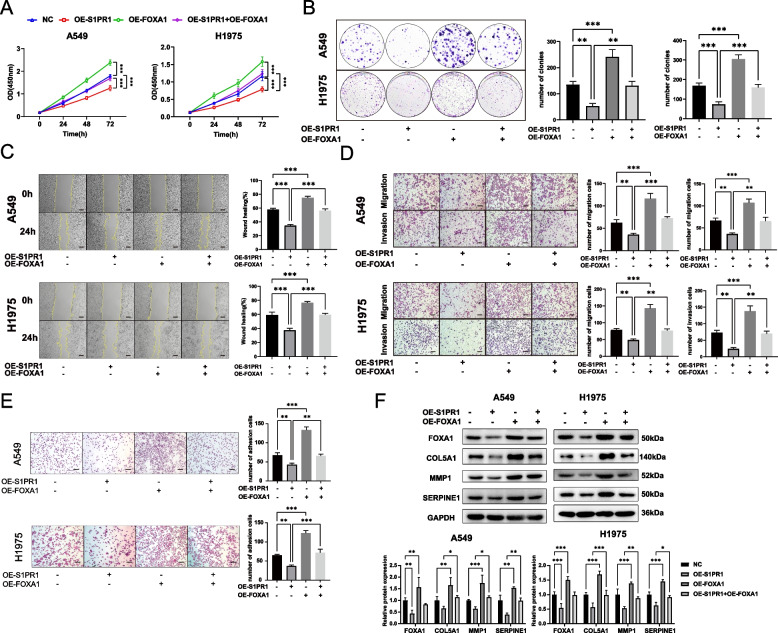
FOXA1 reversed the inhibitory effect of S1PR1 on LUAD cells in vitro. **A** Effect of S1PR1 and FOXA1 on the proliferation ability of A549 and H1975 cells by CCK-8 assay. **B** Effect of S1PR1 and FOXA1 on the colony formation ability of A549 and H1975 cell. **C** Effect of S1PR1 and FOXA1 on the migration ability of A549 and H1975 cells by wound healing assay. Scale bar = 300 μm. **D** Effect of S1PR1 and FOXA1 on the migration and invasive ability of A549 and H1975 cells by transwell migration and invasive assay. Scale bar = 150 μm. **E** Effect of S1PR1 and FOXA1 on the adhesion ability of A549 and H1975 cells by adhesion assay. Scale bar = 150 μm. **F** Effect of S1PR1 and FOXA1 on the expression of COL5A1, MMP1 and SERPINE1 by western blot. ns, not significant, *P < 0.05, **P < 0.01 and ***P < 0.001

To explore the potential role of FOXA1 in mitigating the inhibitory effects of S1PR1 on LUAD cells, we transfected FOXA1 plasmids into A549 and H1975 cells that were engineered to overexpress S1PR1. CCK-8 assay and colony formation assay demonstrated that FOXA1 significantly augmented the proliferative capacity of A549 and H1975 cells, and reversed the suppressive effect of S1PR1(Fig. 5A-B). Wound healing assay and transwell migration assay showed that FOXA1 overexpression promoted the migration potential of A549 and H1975 cells, and mitigated the inhibitory effect imposed by S1PR1 (Fig. 5C-D). Similarly, the transwell invasion assay revealed a significant enhancement in invasive capacity of A549 and H1975 cells upon FOXA1 overexpression, which counteracted the suppressive effect of S1PR1 (Fig. 5D). Furthermore, the adhesion assay demonstrated that FOXA1 overexpression led to a marked enhancement in the adhesive ability of A549 and H1975 cells, while this elevation also abrogated the inhibitory impact of S1PR1(Fig. 5E).

Subsequently, we assessed the protein expression alterations of COL5A1, MMP1, and SERPINE1 via western blot. S1PR1 overexpression led to a reduction in the protein levels of COL5A1, MMP1, and SERPINE1, whereas FOXA1 overexpression increased their expression. Notably, FOXA1 mitigated the decrement in COL5A1, MMP1, and SERPINE1 protein expression induced by S1PR1 overexpression (Fig. 5F).

**Fig. 6:**
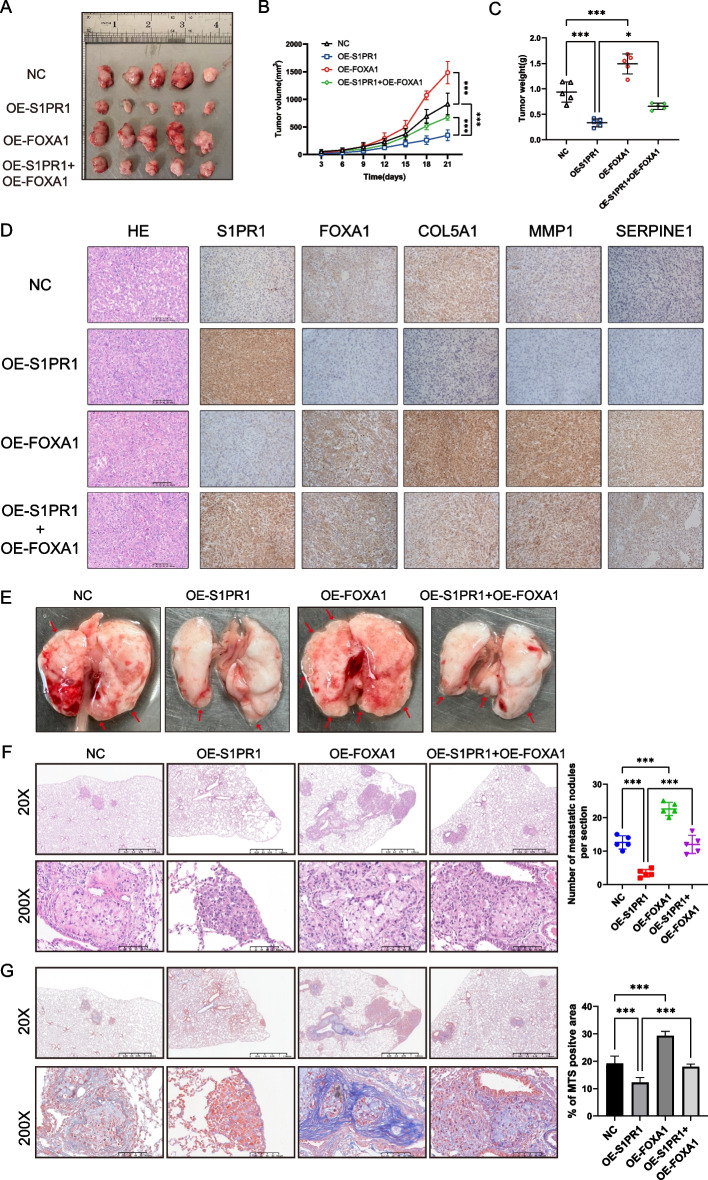
FOXA1 reversed the inhibitory effect of S1PR1 on LUAD cells in vivo. **A** Effect of S1PR1 and FOXA1 on the tumor formation of xenograft in nude mice (*n* = 5). **B** Tumor volumes of xenograft tumors which were measured every 3 days. **C** Average weight of tumors in the end of experiment. **D** Representative H&E and IHC images of S1PR1, FOXA1, COL5A1, MMP1 and SERPINE1 in xenografts from each group. **E** Representative images of lung tissues in lung metastatic models (*n* = 5). **F** Representative H&E staining and statistical analysis on number of lung metastatic nodules (*n* = 5). **G** Representative MTS staining and statistical analysis of lung metastatic models (*n* = 5). *P < 0.05 and ***P < 0.001

To clarify the role of S1PR1 and FOXA1 in tumor growth, we established an in vivo subcutaneous xenograft model. In line with our in vitro results, S1PR1 significantly reduced the volume and weight of subcutaneous tumors compared to the control group. Conversely, FOXA1 overexpression significantly increased the volume and weight of the tumors. Moreover, the inhibitory effect of S1PR1 on tumor growth was attenuated by FOXA1 overexpression, suggesting that the role of FOXA1 in mediating the effects of S1PR1 on lung adenocarcinoma cell proliferation (Fig. 6A-C). IHC revealed that S1PR1 suppressed the expression of FOXA1, COL5A1, MMP1, and SERPINE1, and FOXA1 was able to counteract the inhibitory effect of S1PR1 on these proteins (Fig. 6D). Masson’s trichrome staining was employed to assess the impact of S1PR1 and FOXA1 on collagen fibers in subcutaneous xenograft tumors. The results indicated that overexpression of S1PR1 significantly reduced collagen deposition within the tumors. Conversely, FOXA1 was found to promote abnormal collagen synthesis and deposition, thereby reversing the effects of S1PR1 on collagen (Figure S5).

To investigate the effects of S1PR1 and FOXA1 on lung metastasis, the lung metastasis models were established. Eight weeks after tail vein injection, lung metastasis was observed in all four groups (Fig. 6E). H&E staining confirmed that the number of metastatic nodules was significantly lower in the S1PR1 overexpression group compared to the control group, while more metastatic nodules were observed in the FOXA1 overexpression group. Furthermore, FOXA1 was found to reverse the inhibitory effect of S1PR1 on metastatic nodules (Fig. 6F). Masson’s trichrome staining showed that overexpression of S1PR1 significantly reduced collagen deposition, while FOXA1 promoted the process and reversed the effect of S1PR1 on collagen deposition (Fig. 6G). These results indicate that S1PR1 suppressed lung cancer metastasis through FOXA1 in mouse lung metastasis model.

Collectively, the in vivo studies showed that S1PR1 suppressed the function of LUAD by inhibiting the expression of COL5A1, MMP1, and SERPINE1 through FOXA1, ultimately leading to functional alterations in LUAD.

### S1PR1 regulates FOXA1 through miR-30c-5p

**Fig. 7:**
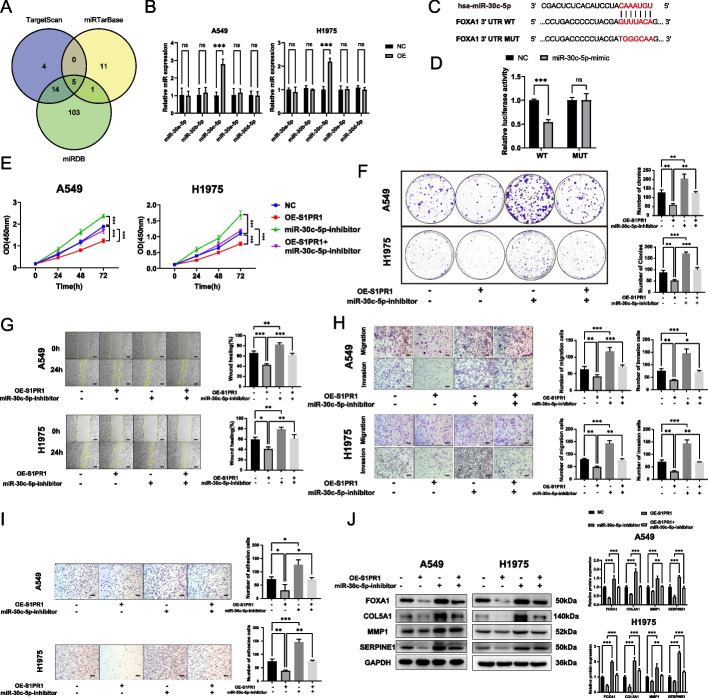
S1PR1 regulates FOXA1 through miR-30c-5p. **A** miRNAs that regulates FOXA1 using Targetscan, miRTarbase, and miRDB databases. **B** miRNA levels of miR-30 family detected by RT-qPCR in A549 and H1975 cells with S1PR1 overexpression. **C** The putative binding sites of miR-30c-5p with FOXA1. **D** The luciferase activity of miR-30c-5p in 293 T cells co-transfected with FOXA1 or FOXA1 mutant plasmid. **E** Effect of S1PR1 and miR-30c-5p inhibitor on the proliferation ability of A549 and H1975 cells by CCK-8 assay. **F** Effect of S1PR1 and miR-30c-5p inhibitor on the colony formation ability of A549 and H1975 cell. **G** Effect of S1PR1 and miR-30c-5p inhibitor on the migration ability of A549 and H1975 cells by wound healing assay. Scale bar = 300 μm. **H** Effect of S1PR1 and miR-30c-5p inhibitor on the migration and invasive ability of A549 and H1975 cells by transwell migration and invasive assay. Scale bar = 150 μm. **I** Effect of S1PR1 and miR-30c-5p inhibitor on the adhesion ability of A549 and H1975 cells by adhesion assay. Scale bar = 150 μm. **J** Effect of S1PR1 and miR-30c-5p inhibitor on the expression of COL5A1, MMP1 and SERPINE1 detected by western blot. ns, not significant, *P < 0.05, **P < 0.01 and ***P < 0.001

To further elucidate the underlying mechanism of S1PR1’s regulation of FOXA1, we conducted a comprehensive search using Targetscan, miRTarbase, and miRDB databases to identify the microRNAs (miRNAs) that modulate FOXA1 in lung cancer tissues or cells. Five miRNAs belonging to the miR-30 family regulated FOXA1, specifically miR-30a-5p, miR-30b-5p, miR-30c-5p, miR-30d-5p, and miR-30e-5p (Fig. 7A). RT-qPCR demonstrated a significant upregulation of miR-30c-5p expression in S1PR1-overexpressing cells compared to control cells. However, no significant differences were observed in the levels of miR-30a-5p, miR-30b-5p, miR-30d-5p, and miR-30e-5p (Fig. 7B). Based on these findings, we posit that S1PR1 regulates the expression of FOXA1 by upregulating miR-30c-5p.

miRNAs play a pivotal role in post-transcriptional regulation of genes through binding to specific sequences in the 3’untranslated region (3’UTR) of target mRNAs, triggering mRNA degradation and thereby inhibiting the expression of the targeted genes. Utilizing Targetscan and miRTarbase databases, we found that miR-30c-5p exhibits complementarity to the base sequence located at positions 432–438 of the FOXA1 3’UTR (Fig. 7C).

To validate this interaction, we employed a dual-luciferase reporter assay. miR-30c-5p significantly enhanced the luciferase activity of a wild-type FOXA1 3’UTR reporter, but did not alter the luciferase activity of a mutant FOXA1 3’UTR reporter, confirming the specificity of the miR-30c-5p-FOXA1 3’UTR interaction (Fig. 7D). Collectively, our findings indicate that miR-30c-5p triggers the degradation of FOXA1 mRNA, ultimately suppressing the malignant biological function of LUAD cells.

To further investigate this mechanism, we treated A549 and H1975 cells overexpressing S1PR1 with a miR-30c-5p inhibitor. CCK-8 assay (Fig. 7E) and colony formation assay (Fig. 7F) revealed that inhibition of miR-30c-5p promoted the proliferation ability of LUAD cells and reversed the suppressive effects of S1PR1 on proliferation. Wound healing assay and transwell migration assay revealed inhibition of miR-30c-5p augmented the migration potential into LUAD cells and restored their migration capabilities after S1PR1 overexpression (Fig. 7G-H). The transwell invasion assay further corroborated these findings, demonstrating inhibition of miR-30c-5p was able to reverse the suppressive effects of S1PR1 on invasion ability (Fig. 7H). In addition, the cell adhesion assay showed miR-30c-5p-inhibitor enhanced the adhesive ability of LUAD cells and reversed the inhibitory effect of S1PR1 (Fig. 7J).

Western blot revealed miR-30c-5p inhibitor induced a significant upregulation in the protein level of FOXA1 and then increased the expression of COL5A1, MMP1, and SERPINE1. Furthermore, the miR-30c-5p inhibitor reverse the suppressive effect of S1PR1 overexpression on FOXA1 and its downstream proteins (Fig. 7J). Based on these findings, we postulate that S1PR1 inhibits the expression of FOXA1 by upregulating miR-30c-5p, thereby suppressing progression of LUAD.

### S1PR1 regulates miR-30c-5p through p-STAT1

**Fig. 8:**
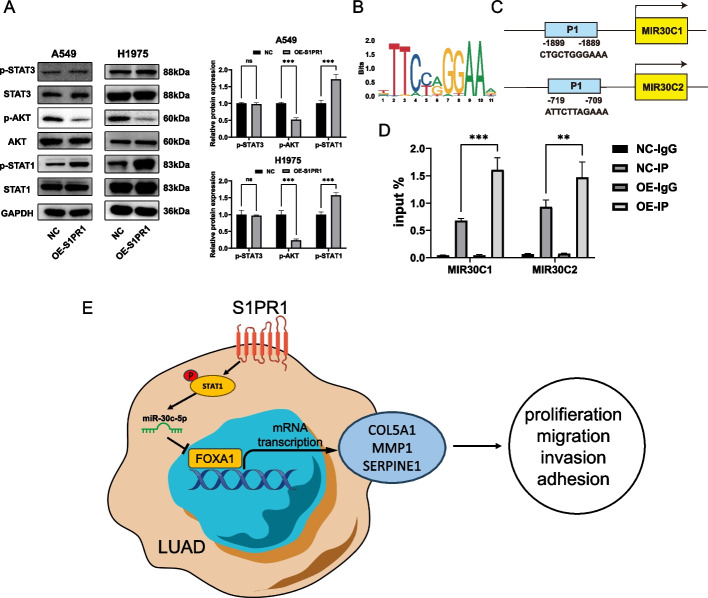
S1PR1 regulates miR-30c-5p through pSTAT1. **A** Protein levels of pSTAT3, STAT3, p-AKT, AKT, p-STAT1, STAT1 detected by western blot in A549 and H1975 cells with S1PR1 overexpression. **B** The motif of STAT1 predicted by JASPAR database. **C** The potential binding sites of STAT1 in promoters of MIR30C1 and MIR30C2 were predicted by JASPAR database. **D** ChIP -qPCR of FOXA1 binding to promoters of MIR30C1 and MIR30C2 in A549 cells with S1PR1 overexpression. **E** Proposed mechanism for a role of S1PR1 in LUAD. ns, not significant, **P < 0.01 and ***P < 0.001

miRNAs are also regulated by similar mechanisms as other mRNAs, such as transcriptional activation or repression [21, 22], epigenetic suppression, and controlled degradation rates [23]. Previous studies have demonstrated that S1PR1 can activate various signaling pathways, including PI3K/AKT, RhoA/ROCK1, β-catenin, and STAT3. We further investigated the changes in the protein expression in LUAD cells by modulating S1PR1. The results demonstrated a significant reduction in p-AKT levels following S1PR1 overexpression in A549 and H1975 cells, whereas p-AKT was upregulated after S1PR1 knockdown in H1975 cells. (Fig. 8A, Figure S6). Previous research has indicated that MMP1 and SERPINE1 can activate PI3K/AKT phosphorylation, promoting tumor progression [24, 25]. Therefore, we speculate that S1PR1 inhibits the expression of MMP1, and SERPINE1, thereby reducing the phosphorylation level of AKT. Additionally, overexpression of S1PR1 resulted in increasing of p-STAT1 in A549 and H1975 cells and S1PR1 knockdown led to p-STAT1 decrease in H1975 cells, while there was no significant difference in p-STAT3 levels (Fig. 8A, Figure S6). Based on these findings, we hypothesize that S1PR1 promotes the transcription of miR-30c-5p by enhancing the phosphorylation of STAT1.

miR-30c-5p possesses two precursors: miR-30c-1, which originates from the transcription of MIR30C1, and miR-30c-2, transcribed from MIR30C2. we explored potential binding sites of STAT1 on the promoter regions of MIR30C1 and MIR30C2 using the JASPAR database. STAT1 indeed possesses the ability to bind to the promoter regions of both MIR30C1 and MIR30C2(Fig. 8B-C). ChIP assay confirmed that STAT1 binds to the promoter regions of MIR30C1 and MIR30C2, thereby regulating their transcription. Moreover, overexpression of S1PR1 induced a significant increase in STAT1 levels in the promoter of MIR30C1 and MIR30C2 (Fig. 8D).

In conclusion, we proposed the following mechanism (Fig. 8E). S1PR1 promoted the transcription of miR-30c-5p via p-STAT1, leading to the suppression of FOXA1. This downregulation subsequently decreases the expression of COL5A1, MMP1, and SERPINE1, ultimately inhibiting the malignant biological function of LUAD.

## Discussion

In this study, we discovered that the expression of S1PR1 is significantly downregulated in LUAD, and this downregulation is strongly correlated with adverse clinical outcomes. We further elucidated the mechanism of S1PR1’s suppressive effect on COL5A1, MMP1, and SERPINE1 via the p-STAT1/miR-30c-5p/FOXA1 signaling pathway, thereby inhibiting specific malignant biological functions of LUAD.

In physiological conditions, S1PR1 is intricately linked to the development and stability of pulmonary microvasculature, playing a pivotal role in maintaining vascular integrity and permeability. S1P has been shown to enhance the barrier function of endothelial cells, and the utilization of the glutathione peroxidase 4 inhibitor RSL3, which induces S1PR1 degradation and internalization, leads to increased endothelial permeability [26]. Conditional ablation of S1PR1 in pulmonary vascular endothelial cells results in heightened endothelial permeability, whereas transplantation of S1PR1-expressing endothelial cells into injured pulmonary vessels restores endothelial integrity [27]. Additionally, S1PR1 expression is reduced in pulmonary microvascular endothelial cells isolated from chronic smokers compared to non-smokers [28], suggesting that S1PR1 deficiency may contribute to the development of lung cancer, given that smoking is a well-established risk factor for this malignancy.

Utilizing database analysis and tissue sample evaluation, we initially verified that S1PR1 is downregulated in lung cancer and is closely associated with patient prognosis. Furthermore, our findings indicate that S1PR1 may be implicated in the functional alterations related to ECM. The role of S1PR1 in enhancing the malignant potential of tumor cells is well-documented in various malignancies. A pivotal mechanism underlying this enhancement involves the activation of the STAT3 signaling pathway by S1PR1, thereby fostering cancer initiation, growth, and dissemination [29]. S1PR1 has been reported to be upregulated by ApoM overexpression, which promotes proliferation and invasion in vitro as well as tumor growth in vivo in the A549 cell line. However, the study did not further modulate S1PR1 to investigate its specific impact on A549 cell function [30]. Notably, our findings demonstrate an intriguing divergence in this paradigm, specifically in LUAD cells. We observed that S1PR1 overexpression did not enhance, but instead inhibited, the malignant biological properties of these cells.

Moreover, some studies have reported contrasting findings in other cancers. Specifically, the downregulation of S1PR1 expression was associated with increased cell migration and invasion, while the upregulation of S1PR1 significantly reduced these malignant behaviors [31]. Furthermore, in the context of bladder cancer, S1PR1 exhibits inhibitory effects on cell migration by suppressing epithelial-mesenchymal transition (EMT). Conversely, the use of FTY720 as an S1PR1 antagonist promotes EMT in bladder cancer cells [32]. Collectively, these observations indicate that S1P may exert diametrically opposed effects in different cancer types, depending on the signaling pathways it modulates.

Utilizing transcriptome sequencing analysis, we pinpointed COL5A1, MMP1, and SERPINE1 as the cardinal target proteins of S1PR1 in the modulation of ECM. Extensive research has revealed that COL5A1 is overexpressed in numerous malignancies, playing a crucial role in fostering tumor progression, conferring resistance to chemotherapy, and regulating the immune microenvironment [33–35]. In the context of LUAD, COL5A1 assumes a pivotal function in facilitating metastasis. Specifically, the downregulation of COL5A1 attenuates the proliferation and invasiveness of LUAD cells, while also inducing apoptosis [36]. MMPs, particularly MMP1, are essential mediators in tissue remodeling, capable of degrading a diverse array of ECM components, thereby fostering cancer cell invasion and metastasis. Aberrant regulation of MMPs is frequently observed in cancer, where they promote tumor progression by remodeling the ECM [37]. MMP1 has been established as a significant biomarker for tumor progression and metastasis across various cancer types, including lung cancer. SERPINE1, on the other hand, facilitates cancer progression, modulates metabolic changes, metastasis, and treatment resistance [38]. In LUAD, SERPINE1 assumes a pivotal role. It promotes EMT by regulating TGF-β signaling, thereby advancing cancer metastasis and invasion [39].

Subsequently, our study revealed that S1PR1 potently suppressed the expression of the transcription factor FOXA1, also referred to as HNF-3α. As a pivotal transcriptional regulator, FOXA1 facilitates the transcription of COL5A1, MMP1, and SERPINE1 by binding to specific promoter regions. Notably, by modulating FOXA1 expression, we successfully reversed the inhibitory impact of S1PR1 on the oncogenic properties of LUAD cells. FOXA1, belonging to the forkhead family of transcription factors, is characterized by its unique wing helix structure and plays a crucial role in epithelial cell differentiation and development across various organs, such as the prostate, and breast [40].

In LUAD, FOXA1 functions as an oncogenic factor through diverse mechanisms, encompassing EMT, non-coding RNA regulation, transcription factor interactions, and cell metabolism. Specifically, FOXA1 contributes to the loss of epithelial characteristics and enhances the invasion and metastasis of lung cancer cells by regulating the expression of ECM-related genes [41]. Additionally, it promotes the proliferation and malignant progression of lung cancer cells by upregulating the expression of DSCAM-AS1 [42]. Moreover, the functionality of FoxA1 is influenced by differential NKX2-1 expression [43, 44]. our findings highlight the significance of the S1PR1-FOXA1 axis in LUAD progression and provide potential therapeutic targets for the disease.

After a thorough analysis, we delved into the intricate mechanism underlying the regulation of FOXA1 expression reduction by S1PR1. Our findings revealed that S1PR1 modulates miR-30c-5p expression by augmenting the phosphorylation status of STAT1. Specifically, miR-30c-5p recognizes and binds to a distinct sequence in the’3’UTR of FOXA1 mRNA, thereby suppressing its transcriptional activity. This cascade of events ultimately attenuates the malignant biological functions of LUAD cells.

miR-30c-5p has garnered significant attention due to its robust tumor-suppressing capabilities across a range of malignancies. In the context of lung epithelial cells, it impedes autophagy by targeting CTGF and ATG5, effectively hampering the EMT process, a crucial step in silicotic fibrosis inhibition [45]. Moreover, in endometrial cancer, miR-30c executes its tumor-suppressive role by targeting MTA1, thereby curtailing cancer cell proliferation, migration, and invasion [46]. In cervical cancer, miR-30c-5p targets the METTL3/KRAS axis, fostering ferroptosis and concomitantly suppressing tumor growth and metastasis [47]. Additionally, miR-30c delivered by bone marrow mesenchymal stem cells has been shown to induce apoptosis and attenuate the migration and invasive capacities of glioblastoma cells [48].

STAT1, a member of the signal transducer and activator of transcription (STAT) protein family, is widely recognized as a tumor suppressor. Its elevated expression has been associated with the suppression of stemness characteristics in paclitaxel-resistant ovarian cancer cells [49]. Furthermore, STAT1 inhibits the expression of FOXM1 in pancreatic cancer cells, thereby promoting gemcitabine-induced apoptosis [50]. Concurrently, downregulation of STAT1 signaling leads to a reduction in NQO1 expression, which enhances oxidative stress and ultimately augments the cytotoxic effects of phenformin on breast cancer cells [51].Our investigation uncovered that S1PR1 enhances the binding affinity of STAT1 to the promoter regions of MIR30C1 and MIR30C2, leading to an upregulation of miR-30c-5p expression. This, in turn, suppresses the expression of FOXA1, ultimately inhibiting the malignant phenotype of LUAD cells.

## Conclusions

S1PR1 is downregulated in LUAD, which is correlated with poor prognosis. S1PR1 regulates the malignant function of LUAD cells by inhibiting the expression of COL5A1, MMP1 and SERPINE1 through the p-STAT1/miR-30c-5p/FOXA1 signaling pathway.

## Supplementary Information

Supplementary Material 1.

Supplementary Material 2.

## Data Availability

All data generated during this study are available from the corresponding author on reasonable request.

## Abbreviations

CC: Cellular Component
CCK-8: Cell Counting Kit-8
ChIP: Chromatin Immunoprecipitation
DEG: Differential Expression Gene
ECM: Extracellular matrix
FBS: Fetal bovine serum
GEO: Gene Expression Omnibus
GO: Gene Ontology
GSEA: Gene set enrichment analysis
IHC: Immunohistochemistry
KEGG: Kyoto Encyclopedia of Genes and Genomes
LUAD: Lung adenocarcinoma
MF: Molecular function
miRNA: MicroRNA
NC: Negative Control
OE: Overexpression
PCR: Polymerase Chain Reaction
RT-qPCR: Real time-quantitative polymerase chain reaction
S1P: Sphingosine-1-phosphate
S1PR: Sphingosine-1-phosphate receptor
shRNA: Short hairpin RNA
TCGA: The Cancer Genome Atlas
