# Supplementary material for: S1PR1 suppresses lung adenocarcinoma progression through p-STAT1/miR-30c-5 p/FOXA1 pathway
Source: J Exp Clin Cancer Res. 2024 Nov 18;43:304. doi: 10.1186/s13046-024-03230-5 (PMC11571582; doi:10.1186/s13046-024-03230-5)
Supplement: Supplementary file 2 — Supplementary Material 2. [file 13046_2024_3230_MOESM2_ESM.pdf]

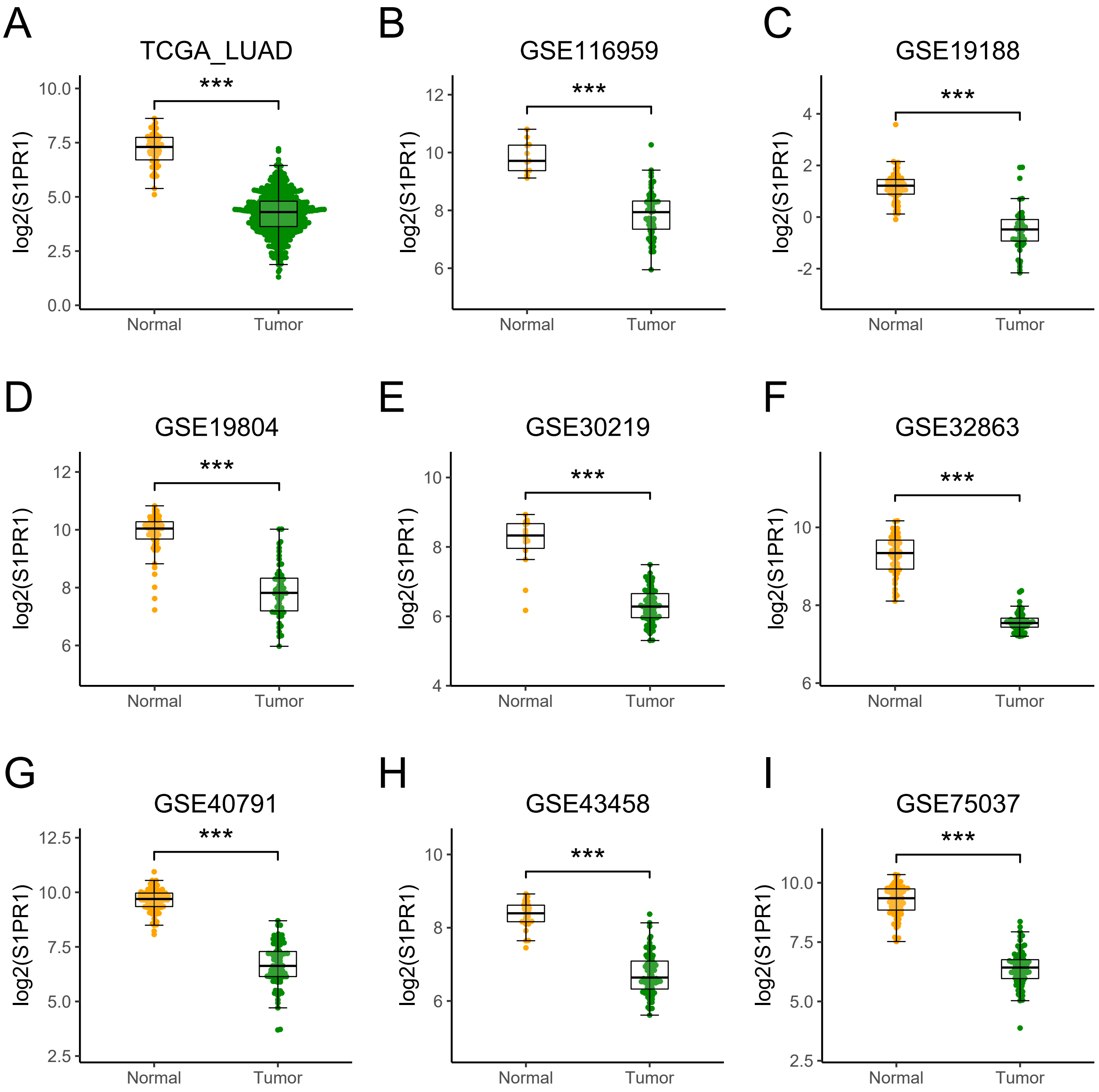

Figure S1. The difference of S1PR1 expression between normal and tumor tissues in LUAD patients. （A）TCGA LUAD,（B）GSE116959,（C）GSE19118,（D）GSE19804,（E）GSE30219,（F）GSE32863,（G）GSE40791,（H）GSE43458,（I）GSE75037. ***P<0.001.

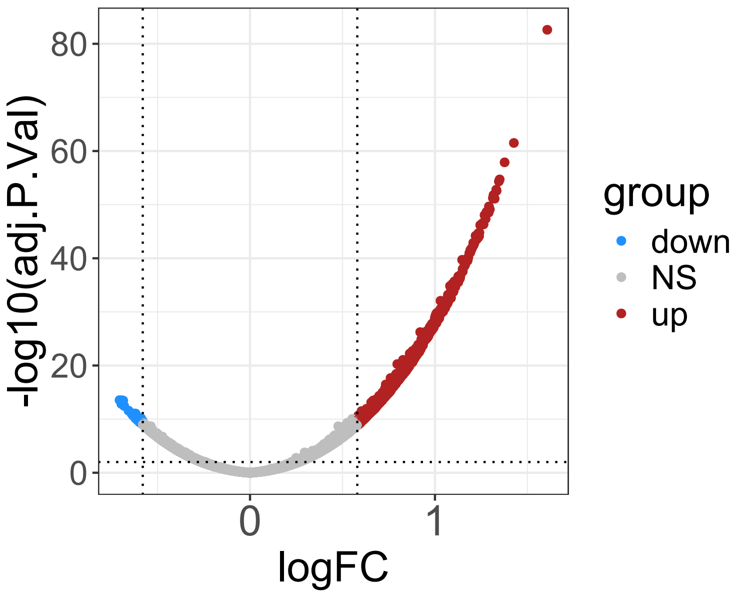

Figure S2. DEGs between high and low S1PR1 groups in lung adenocarcinoma from the TCGA database.

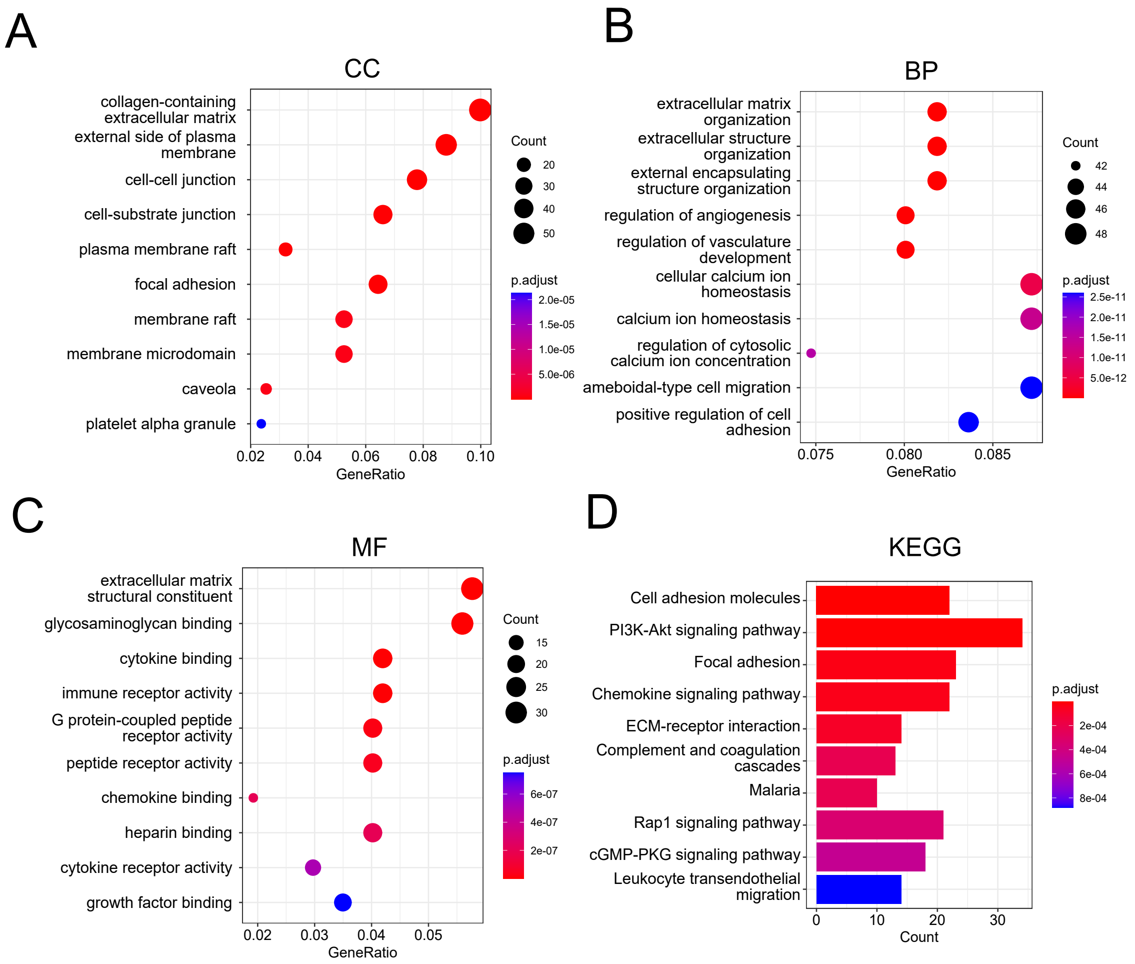

Figure S3. GO function and KEGG pathway enrichment analysis of the DEGs. （A）cellular component(CC),（B）biological process(BP),（C）molecular function(MF),（D）KEGG.

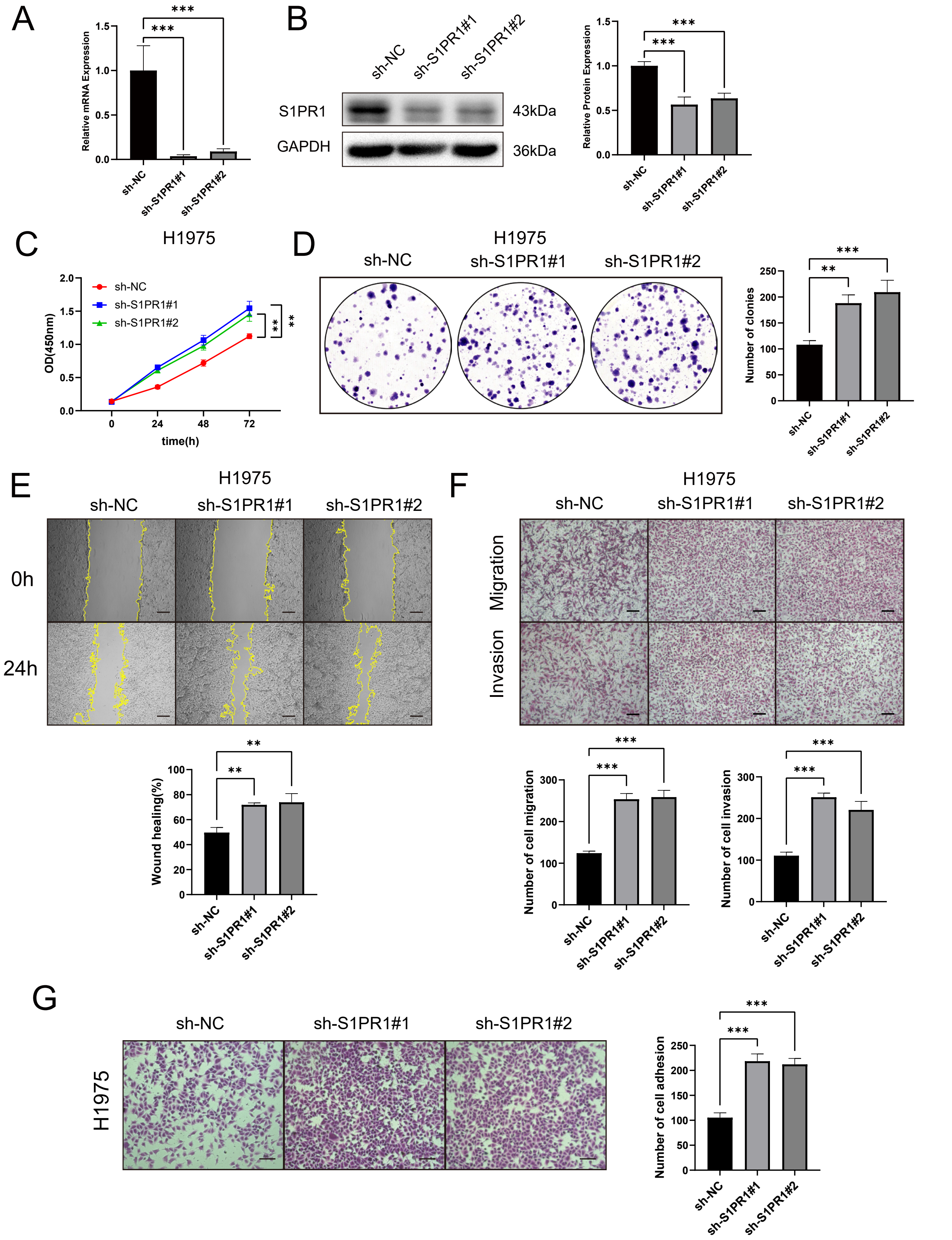

Figure S4. Knockdown of S1PR1 promotes the proliferation, migration, invasion and adhesion of LUAD cells. **(A)** mRNA expression levels and **(B)** protein levels of S1PR1 in H1975 cells after S1PR1 knockdown. **(C)** Effect of S1PR1 knockdown on the proliferation ability of H1975 cells by CCK-8 assay. **(D)** Effect of S1PR1 knockdown on the colonies formation of H1975 cells by colony formation assay. **(E)** Wound healing assay showed S1PR1 knockdown promoted migration ability of H1975 cells. Scale bar =300μm. **(F)** Effect of S1PR1 knockdown on the migration and invasive ability of H1975 cells by transwell migration and invasive assay. Scale bar =150μm. **(G)** Effect of S1PR1 knockdown on the adhesion ability of H1975 cells by adhesion assay. Scale bar =150μm. $^{**}p < 0.01$ and $^{***}p < 0.001$.

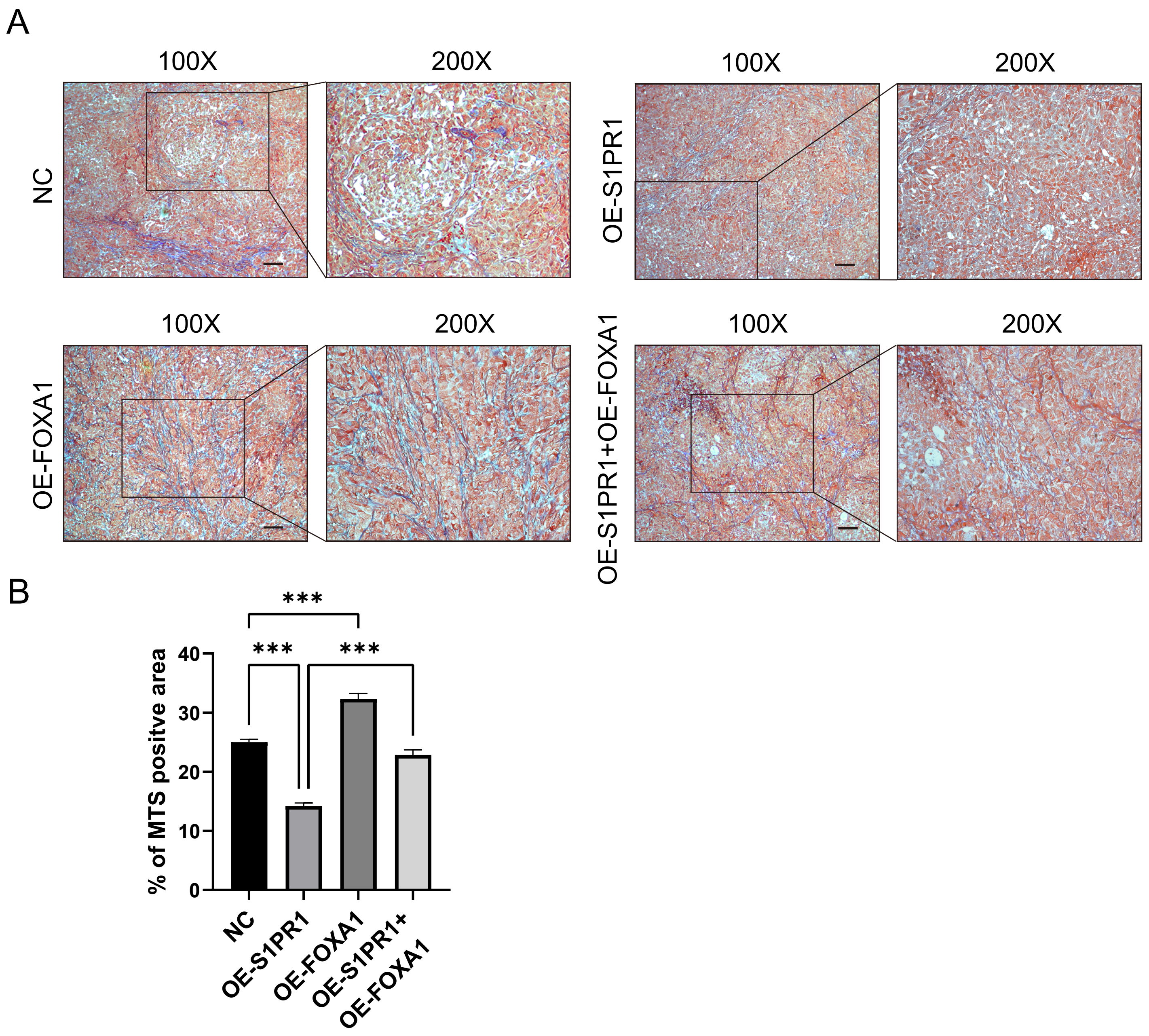

Figure S5. Effects of S1PR1 and FOXA1 on collagen fibers in subcutaneous xenograft tumors.
(A) Representative Masson's trichrome staining(MTS) of subcutaneous xenograft tumors. Scale bar, 100 μm.
(B) Quantification of the percentage of MTS area. ***p < 0.001

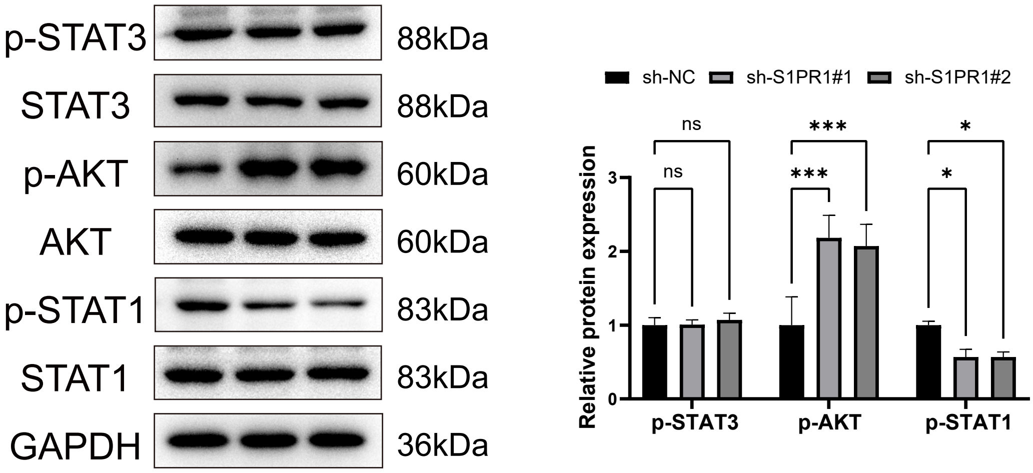

Figure S6. Protein levels of p-STAT3, STAT3, p-AKT, AKT, p-STAT1, STAT1 detected by western blot in H1975 cells with S1PR1 knockdown. ns, not significant, *p<0.05, and ***p < 0.001.
